# The glycolytic process in endothelial cells and its implications

**DOI:** 10.1038/s41401-021-00647-y

**Published:** 2021-04-13

**Authors:** Susan, Wai Sum Leung, Yi Shi

**Affiliations:** 1 Department of Pharmacology and Pharmacy, Li Ka Shing Faculty of Medicine, The University of Hong Kong, Hong Kong SAR, China; 2 Institute of Clinical Science, Zhongshan Hospital, Fudan University, Shanghai, 200032 China; 3 Key Laboratory of Organ Transplantation, Zhongshan Hospital, Fudan University, Shanghai, 200032 China

**Keywords:** endothelial cells, glycolysis, glucose transporters, hexokinase 2, phosphofructokinase/fructose bisphosphatase 3, pyruvate kinase enzyme M2, cardiovascular disease

## Abstract

Endothelial cells play an obligatory role in regulating local vascular tone and maintaining homeostasis in vascular biology. Cell metabolism, converting food to energy in organisms, is the primary self-sustaining mechanism for cell proliferation and reproduction, structure maintenance, and fight-or-flight responses to stimuli. Four major metabolic processes take place in the energy-producing process, including glycolysis, oxidative phosphorylation, glutamine metabolism, and fatty acid oxidation. Among them, glycolysis is the primary energy-producing mechanism in endothelial cells. The present review focused on glycolysis in endothelial cells under both physiological and pathological conditions. Since the switches among metabolic processes precede the functional changes and disease developments, some prophylactic and/or therapeutic strategies concerning the role of glycolysis in cardiovascular disease are discussed.

## Introduction

The endothelium lies on the innermost layer of blood vessels, regulating local vascular tone and permeability and coordinating with neighboring cells to modulate immune/inflammatory responses and blood supply. Endothelial dysfunction is a critical and initiating factor in the development of cardiovascular disease [1]. In blood vessels, endothelial nitric oxide synthase (eNOS, NOS III) is constitutively activated upon laminar flow. Nitric oxide (NO) is the primary regulator of local vascular tone and inhibits other vasoactive factors. When the protective effects of NO recede, protein expressions of adhesion molecules are increased in endothelial cells, thus promoting platelet aggregation and leukocyte adhesion [1, 2]. It is of importance that endothelial cells are highly heterogeneous depending on their location and microenvironments [2–7].

Cell metabolism, converting food to energy in organisms, is the primary self-sustaining mechanism for cell proliferation and reproduction, structure maintenance, and fight-or-flight responses to stimuli. Four major metabolic processes take place in the energy-producing process, including glycolysis, oxidative phosphorylation, glutamine metabolism, and fatty acid oxidation [8]. The metabolic changes respond quickly to physiological and pathological stimulations, including hypoxia, inflammation, and immune stimulation [9]. More importantly, the switch of metabolic processes precedes functional changes and disease developments [9, 10]. Endothelial cells, in both macro- and microcirculation, produce adenosine triphosphate (ATP) mainly by glycolysis, which is comparable with cancer cells and neutrophils [11–13]. Most cells, including cancer cells, generate ATP via the oxidative phosphorylation in the tricarboxylic acid cycle (TCA), but switch to glycolytic ATP production under hypoxia conditions. Unlike cancer cells, endothelial cells are exposed to the highest oxygen levels and produce ATP via aerobic glycolysis. It is assumed that endothelial cells (1) facilitate oxygen diffusion to perivascular cells by using minimal oxygen, (2) reduce reactive oxygen species generation by preferentially using glycolysis [14]. Thus, the present review focuses on endothelial glycolysis under physiological and pathological conditions.

## Glycolysis

**Fig. 1:**
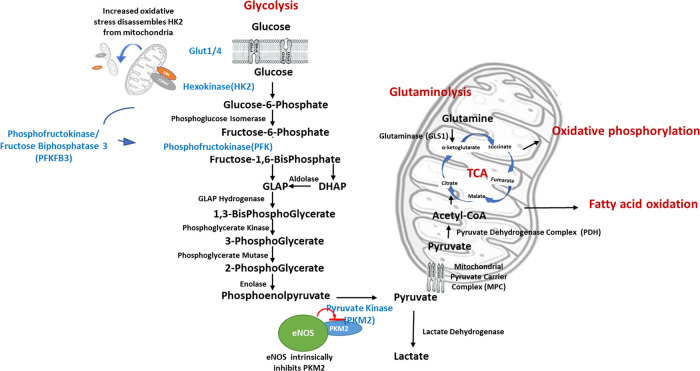
Interaction of glycolysis, oxidative phosphorylation, glutaminolysis, and fatty acid oxidation. More than ten enzymes participate in the glycolysis process, transferring glucose to lactate and producing ATP molecules, including the glucose transporters (GLUT1 and GLLUT4), HK2, PFK, and PKM2. Products from the pyruvate kinase go either into the TCA cycle under aerobic conditions or to lactic acid under anaerobic conditions. PFKFB3 is an allosteric activator of PFK and plays a crucial role in regulating the glycolytic process. MPC transports pyruvate in the cytosol across the inner mitochondrial membrane. HK2 protein locates at the outer mitochondrial membrane. Increased mitochondrial fission separates HK2 molecules from mitochondria, resulting in mitochondrial permeability transition pore opening and increased oxidative stress. Glutamine metabolism deaminates glutamine to glutamate by GLS1, serves as a critical process in replenishing the carbon intermediates for maintaining the mitochondrial TCA cycle. PKM2 protein is part of the eNOS complex, and eNOS has intrinsic inhibitory effects on PKM2 activity through nitrosation under control conditions.

More than ten enzymes participate in the glycolysis process, transferring glucose to lactate and producing ATP molecules (Fig. 1). The glucose transporters (GLUT1 and GLUT4), also known as solute carrier family 2 members, are responsible for taking up extracellular glucose into cells. The contribution of GLUT1 and GLUT4 to glucose transport in endothelial cells depends on the pathological conditions investigated, the experimental model designed, vascular beds involved, and the stimuli applied. Hexokinase 2 (HK2), the first rate-limiting enzyme in the process, catalyzes glucose to glucose-6-phosphate. Phosphofructokinase (PFK) is the second rate-limiting enzyme, converting fructose-6-phosphate into fructose 1,6-bisphosphate and adenosine diphosphate (ADP). PFK/fructose bisphosphatase 3 (PFKFB3) is an allosteric activator of PFK. As such, PFKFB3 plays a crucial role in regulating the glycolytic process, especially under pathological conditions [12], and hence in regulating angiogenesis [15, 16]. The pyruvate kinase enzyme M2 isoform (PKM2) is the third and final rate-limiting enzyme. PKM2 transfers phosphate from phosphoenolpyruvate to ADP and produces ATP molecule. PKM2 is the fine-tune regulator for cell energy generation since pyruvate, the product from PKM2, goes either into the TCA under aerobic conditions or to lactic acid under anaerobic conditions. Phosphoglycerate kinase, the other ATP-produced enzyme, catalyzes a reversible reaction, shifting towards the generation of ATP and 3-phosphoglycerate in glycolysis and that of ADP and 1,3-bis-phosphoglycerate in gluconeogenesis.

### Glycolysis and other metabolic processes

Glycolysis is not an independent process. Cells switch metabolic process upon their pathophysiological needs. Hypoxia increases glycolysis by downregulating mitochondrial pyruvate carrier complex (MPC) while inhibiting MPC promotes glycolysis by upregulating glycolytic enzymes HK2, PFKFB3, and lactate dehydrogenase [17].

Of note, HK2 is present at the outer mitochondrial membrane where it binds to the voltage-dependent anion channel 1 [18], suggesting that HK2 plays a role in controlling reactive oxygen species generation. Increased mitochondrial fission results in the dissociation of voltage-dependent anion channel 1 and HK2 molecules from mitochondria, and the subsequent opening of mitochondrial permeability transition pore and increased oxidative stress [19]. Preventing HK2 dissociation limits mitochondrial reactive oxygen species in cardiomyocytes [18, 20] and cultured endothelial cells [21, 22] and inhibits apoptosis in cultured human microvascular endothelial cells [23]. Inhibition of PKM2 alters mitochondrial substrate utilization [24] by switching the metabolic pathway to the pentose phosphate pathway in endothelial cells [25]. Pyruvate dehydrogenase (PDH), converting pyruvate to acetyl-coenzyme A for fueling the TCA cycle, promotes the shifting of cytosolic glycolysis to mitochondrial oxidative phosphorylation. In cultured human umbilical vein endothelial cells (HUVEC), inhibition of PDH reduces lactate production per glucose consumption (glycolytic index) and increases the mitochondrial oxygen consumption rate.

Glutamine metabolism (glutaminolysis), which involves the deamination of glutamine to glutamate by the enzyme glutaminase (GLS1), serves as a critical process in replenishing the carbon intermediates for maintaining the mitochondrial TCA cycle [26]. A stiff matrix, which mimics stiffness in hypertensive pulmonary arterioles, activates GLS1 and glycolysis, decreases oxidative phosphorylation, and restores cell proliferation in human pulmonary arterial endothelial cells than a soft matrix (mimicking stiffness in non-diseased pulmonary arterioles in rodents), whereas inhibition of GLS1 reduces glutaminolysis and attenuates cell proliferation [27–29]. Combined inhibition of GLS1 and PDH reduces endothelial proliferation, migration, and tube formation; these effects are coupled with normalized mitochondrial oxidation [27].

Activated Wnt signal increases mitochondrial biogenesis and function, but inhibits glycolysis in endothelial progenitor cells [30].

## Endothelial cell phenotypes and glycolysis

### Glycolysis in tip and stalk cells in the angiogenesis

Compared with quiescent endothelial cells, endothelial cells are activated during angiogenesis for vessel sprouting, changing into the migrating tip cell (with long and dynamic filopodia) and the elongated and proliferative stalk cell (with fewer filopodia than the tip cell and for establishing adherent and tight junctions). Pathological angiogenesis usually displays dilated and tortuous vessels covered with hyper motile endothelial cells [31, 32]. Vascular endothelial growth factor (VEGF) and its corresponding receptors, mainly VEGFR2, are critical signaling in the process [33]. It is stated that the tip cell is more active and responsive to the VEGF signal than stalk cells. Enhanced glycolysis is the crucial mechanism for competitiveness in the tip cells. However, the metabolic status is dynamically interchangeable, allowing cells to overtake each other in sprouting [34, 35]. In cultured HUVEC, glycolysis is essential for tip cell differentiation, whereas both glycolysis and mitochondrial respiration are active for non-tip cell proliferation [36]. Using Seahorse flux analyses, in vitro cultured tip cells display lower glycolysis, but a higher capacity to respond to metabolic stress than non-tip cells [37]. Through integrated computational and experimental approaches, enhanced glycolysis and elevated ATP levels promote endothelial cell rearrangement by increasing filopodia formation and reducing intercellular adhesion. PFKFB3 overexpression overrules the pro-stalk activity of Notch signals [12]. Inhibition of PFKFB3 affects VEGFR2 activation in tip cells and normalizes disorganized angiogenesis in high VEGF conditions [12, 38].

### Glycolysis in the blood–brain barrier endothelial cells

**Fig. 2:**
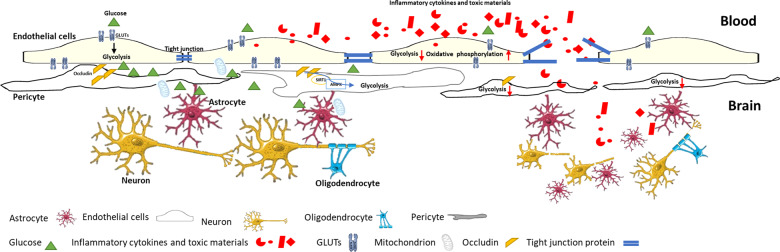
The blood–brain barrier (BBB), consisted of endothelial cells and pericytes, plays a crucial role in protecting the central nervous system from peripheral inflammatory and toxic materials. GLUTs, coupled with HK2 protein in the abluminal side, facilitate the influx of glucose from the blood into the brain. Both BBB endothelial cells and pericytes transfer mitochondria and glucose to astrocytes, providing energy to poor-energic astrocytes under pathophysiological conditions. When the glycolysis is compromised under pathological conditions, endothelial cells switch to other metabolic processes, mainly oxidative phosphorylation, for energy-sustaining, resulting in increased oxidative stress and enhanced inflammation. The latter breaks tight junction and increases the BBB permeability. The presence of occludin in pericytes, also acting as an NADH oxidase and a regulator of AMPK, in the BBB pericyte is depleted under inflammatory conditions, resulting in reduced AMPK signaling and glycolysis process.

The blood–brain barrier (BBB) plays a crucial role in protecting the central nervous system from peripheral inflammatory and toxic materials [39–42]. For this function, brain microvascular endothelial cells display tight junctional structure and low permeability [39–42]. The uptake of glucose into endothelial cells is mediated by GLUTs, which appears to have a higher abundance in the luminal membrane than in the abluminal side in humans [43–45], while the opposite is observed in rodent brains [39, 46]. This inconsistency is probably due to the experimental model designed and the sensitivity of antibodies used. The asymmetrical distribution of GLUTs in BBB endothelial cells, coupled with the presence of HK2 in the abluminal side, creates a concentration gradient of glucose, thereby facilitating the influx of glucose from the blood into the brain [45]. The GLUT1 protein translocates to the luminal membrane during the conditions requiring increased glycolysis [47]. In mice with BBB endothelium-specific, tamoxifen-inducible deletion of GLUT1 protein, mice die in 4 days of tamoxifen-treatment initiation [48]. Further, BBB endothelial cells are more vulnerable to glucose deprivation than the parenchymal cells [49]. Thus, it indicates that the BBB endothelial cells and glucose transporters are critical in regulating glucose transport from the circulatory system to the nervous system [48, 49]. In mice fed with a high-fat diet, GLUT1 protein expression in BBB endothelial cells is downregulated after 3 days; the presence of GLUT1 is slowly restored, accompanied by an increased serum level of VEGF when the high-fat diet is maintained for 1 month [48], indicating that VEGF is a compensatory regulation for GLUT1 regulation in BBB endothelial cells. A reduction of GLUT1 expression in BBB endothelial cells by depletion of VEGF results in accelerated cognitive deficits and increased vascular leakage in a Alzheimer mouse model fed with high-fat diet [47]. Of note, the insulin-responsive glucose transporter GLUT4 colocalizes with GLUT1 and the tight junction proteins zonula and occludins-1 on the BBB endothelial cells in the ventromedial hypothalamus serving as a glucose sensor responsible for initiating the counter regulatory response to hypoglycemia [50] (Fig. 2).

Of importance, the direct effects of reduced glycolysis on increased BBB endothelial permeability have been less studied. Endothelial cells switch to other metabolic processes, mainly oxidative phosphorylation, resulting in increased levels of oxygen-derived free radicals under pathological conditions. Reactive oxygen species, together with byproducts from impaired glycolysis, also generate glycolytic intermediates, for example, advanced glycation end products. Overproduction of ROS results in the activation of endothelial adhesion molecules, such as VCAM-1, to further increase vascular permeability and monocyte adhesion.

### Glycolysis in hematopoietic cells and vascular stem cells

Differentiation of pluripotent stem cells or hematopoietic cells into vascular endothelium is of great importance to angiogenesis and tissue repair [9]. Hematopoietic cells show the low expressions of genes related to glycolysis and the TCA cycle [51, 52]. Enhanced glycolysis in hematopoietic cells promotes endothelial maturation and tube formation and accelerates the development of diabetic retinopathy [52].

## Cell-to-cell communication

### Circulating factors

Cellular small-molecule products are released or permeated to extracellular environments, including the circulatory system. Serum PKM2 level is increased in cancer patients and promotes tumor angiogenesis by increasing endothelial cell proliferation and migration [53]. Plasma 5′-adenosine monophosphate level is increased in type 2 diabetic mice, which stimulates glucose-6-phosphatase enzyme activity and attenuates insulin-dependent GLUT4 translocation in skeletal muscle [54]. Serum from relapsing-remitting multiple sclerosis patients reduces glycolysis, which is associated with the downregulation of membrane GLUT1 expression in cultured brain endothelial cells, leading to enhanced permeability [55].

Exosomes are membrane-bound extracellular vesicles containing various molecular messages, including proteins and RNA. Exosome and its cargo components are essential for message delivery in neighboring and remote communication. Energy depletion by the inhibition of glycolysis and oxidative phosphorylation in endothelial cells stimulates exosome secretion as a result of reduced ATP production coupled with increased 2′-3′-cyclic AMP and AMP levels [56].

### Neighborhood communication

Neutrophils are an important player in cardiovascular inflammation, apart from their crucial role in fighting against bacterial infection. Neutrophils use glycolysis for ATP generation [13]. Lipopolysaccharide (LPS)-induced inflammation enhances glycolytic activities and increases its metabolic byproducts lactate in neutrophils. The released lactate promotes neutrophil mobility by downregulating vascular endothelial-cadherin expression, leading to increased vascular permeability [57].

Endothelial-derived lactate facilitates macrophage to change to a pro-angiogenic and pro-regenerative M2-like phenotype [58, 59]. Deleting PFKFB3 in endothelial cells arrests the M2-like polarization of bone-marrow-derived macrophages, the latter is restored by lactate replenishment [58]. In retinal myeloid cells, particularly macrophages/microglia, the deletion of PFKFB3 prevents them from differentiating into an angiogenic phenotype in the vascular niche in a mouse oxygen-induced proliferative retinopathy model [59]. Blocking glycolysis reduces IL-10 levels in LPS-stimulated macrophages [60]. Therefore, it indicates that glycolysis plays a vital role in the cross talk between endothelial and immune/inflammatory cells.

Pericytes are perivascular cells enveloped in the vascular basement membrane, which is shared with endothelial cells. They are mainly localized in the microvasculature and play a role in vascular development, remodeling, and homeostasis. Disruption of the pericyte–endothelial interaction results in the impairment of angiogenesis. Increased expression of mitochondrial PDK4 in lung pericytes suppresses mitochondrial activity in favor of glycolysis, resulting in pericyte proliferation, reduced endothelial–pericyte interaction, and loss of pulmonary vessels during the progression of pulmonary arterial hypertension [61]. The presence of occludin, also acting as an NADH oxidase and a regulator of monophosphate-activated protein kinase (AMPK), in the BBB pericyte is depleted under inflammatory conditions, resulting in reduced AMPK signaling and glycolysis process, and subsequently reduced glucose uptake and ATP generation in the pericyte as well as reduced glucose transfer to astrocytes [62]. Of note, both pericytes and microvascular endothelial cells transfer mitochondria and glucose to astrocytes, promoting astrocytes’ survival when their energy-producing processes are impaired [62]. Therefore, energy communication in BBB endothelial cells, pericytes, astrocytes, and neurons is critical for neurovascular unit function (Fig. 2).

Hepatic stellate cells from patients with liver fibrosis release exosomes containing GLUT1 and PKM2. By taking up the exosome, liver sinusoidal endothelial cells and Küpffer cells exhibit increased expressions of glycolytic proteins, thus accounting for the increased glycolysis in the process of liver fibrosis [63].

Silent mating type information regulation 2 homolog (sirtuin)3-knockout endothelial cells show activated hypoxia-induced factor 1α (HIF-1α) signaling, upregulated GLUT1 expression as well as glycolysis, and a decreased release of apelin. The decreased level of apelin in turn prevents hypoxia-induced upregulation of GLUT1 and GLUT4 expressions in cardiomyocytes. It implies that endothelial sirtuin 3 regulates cardiomyocyte glucose availability in a paracrine manner [64]. In diabetes, exosomes released from cardiac microvascular endothelial cells are taken up by cardiomyocytes, which disrupts GLUT4 membrane translocation in cardiomyocytes and deteriorates diabetic cardiomyopathy [65].

Transforming growth factor-β stimulates microRNA143/145, which are transferred from smooth muscle cells to endothelial cells, where they downregulate HK2 and integrin β8, respectively [66].

## Signaling pathways involved in the regulation of endothelial glycolytic activities

### Endothelial nitric oxide synthase, nitric oxide, and glycolysis

Endothelial-derived NO is the primary regulator for maintaining local homeostasis. It is reported that PKM2 protein is part of the eNOS complex under basal condition. Moreover, eNOS has intrinsic inhibitory effects on PKM2 activity through nitrosation [25]. Knock-in activated eNOS increases NO-dependent relaxations and shifts cell metabolism to the pentose phosphate pathway by inhibiting PKM2, the latter leads to the production of reducing equivalents NADPH and GSH, thus contributes to antioxidant responses of cultured HUVEC [25].

It is also reported that eNOS, by producing NO, increases glycolysis by increasing the activation of glycolytic enzymes (e.g., GLUTs and PFKFB3) [67–69] and/or AMPK [70] and HIF [71] pathways.

### Shear stress and glycolysis

It has been well-established that shear stress results in activation, through phosphorylation, of eNOS. As such, shear stress may indirectly regulate endothelial glycolysis through NO production. Laminar shear stress inhibits endothelial cell metabolic process via krüppel-like factor 2-mediated PFKFB3 repression in perfused hearts [72]. Of note, oscillatory shear stress significantly upregulates VEGFR-dependent protein kinase C (PKC)ε expression and enhances glycolysis in cultured human aortic endothelial cells [73]. Silencing PKCε reduces endothelial tube formation and vascular repair in a zebrafish model [73]. The enhanced glycolysis restores shear stress-induced NO production in cultured human aortic endothelial cells [74].

However, in atheroprone areas where endothelial cells are exposed to disturbed flow, mainly resembling oscillatory shear stress, AMPK-dependent glycolysis is enhanced and further increases endothelial cell viability and integrity of the endothelial cell barrier. Inhibition of the AMPK pathway reduces endothelial glycolysis and cell viability and accelerates atherosclerosis lesion formation in hyperlipidemic mice [75]. It implies that the activation of AMPK-dependent glycolysis is protective against the development of atherosclerosis.

### Oxidative stress, inflammation, and glycolysis

Increased oxidative stress is a crucial mechanism underlying cardiovascular disease. It is reported that hypoxia increases glycolysis in endothelial cells by upregulating HIF-1α and subsequently GLUT1 and HK2 expression by inhibiting the enzyme NADPH oxidase (Nox) protein. Scavenging reactive oxygen species reverses low oxygen tension-induced metabolic effects [76]. Increased Nox4-dependent production of superoxide anions reduces GLUT4 expression and impairs glycolysis in rat aortic endothelial cells challenged with palmitic acid, a saturated free fatty acid that induces insulin resistance [77]. In rat retinopathy of prematurity model, inhibition of uncoupling protein 2 increases GLUT1 expression and improves physiological retinal vascular development [78]. Nevertheless, it is also reported that hypoxia inhibits the mitochondrial oxidative phosphorylation pathway and shifts ATP production to glycolysis by downregulating MPC, thereby inhibiting the transport of pyruvate to the mitochondrial matrix [24].

Enhanced inflammatory responses disturb glycolysis in endothelial cells. Lipoprotein and its oxidized phospholipids content induce glycolysis by activating PFKFB3 expression and stimulating cell proliferation in cultured human endothelial cells [79, 80]. Pro-inflammatory cytokines also increase glycolysis in human coronary endothelial cells by upregulating glycolytic enzyme expressions, including those of HK2, PKM2, and PFKFB3 [81–83]. Abolishing endothelial PFKFB3 expression protects mice from LPS-induced sepsis [82].

It is recognized that increased oxidative stress and enhanced inflammation are team players in the development of cardiovascular diseases. The controversial results discussed above are probably due to the use of different stimuli, including concentrations and exposure time, and of endothelial cells from different vascular beds. Taken into conjunction, the glycolytic activity in endothelial cells is dynamically regulated by different signaling pathways to support endothelial cell functions during blood vessel formation for development or wound healing or for the maintenance of cell function and integrity.

## Endothelial glycolysis in aging, obesity, and diseases

### Aging

Aging is an inevitable part of life. Aging-related physiological changes resemble those occurring under pathological conditions, including hypertension, diabetes, and atherosclerosis [84–87]. In 24-month old rats, protein presences of GLUT4 are downregulated in the aorta compared with 3-month old rats. Insulin fails to increase GLUT4 and eNOS expressions in primary cultured aortic endothelial cells of aged rats and in the aorta of aged rats [84]. It supports the note that aging is an independent risk factor for cardiovascular disease and metabolic syndrome [88].

### Obesity

Obesity is a severe physical and mental issue in modern society. Obese people are often accompanied by insulin resistance, type 2 diabetes, and chronic inflammation. In obese mice, genetically mutated *ob/ob* mice or high-fat diet-fed mice, pancreatic islet vasculature is dilated, without the occurrence of angiogenesis, to compensate for β-cell hyperplasia and hypertrophy. Mice heterozygous for inactivation of the GLUT4 gene (GLUT4^+/−^) also present with intraislet capillary enlargement, indicating that GLUT4 is not involved in the pathological changes [89].

In line with those mentioned above that VEGF-induced GLUT1 upregulation in BBB endothelial cells [see section: glycolysis in the blood–brain barrier endothelial cells], the increased level of VEGF is released by myeloid cells since specific deletion of VEGF on myeloid cell significantly reduces GLUT1 expression and impairs glucose uptake in BBB endothelial cells, resulting in cognitive deficits. Nevertheless, the protective effects of myeloid cell-derived VEGF are observed in obese mice, but not in lean counterparts, suggesting that obesity-activated macrophages play a homeostatic role in restoring cerebral glucose metabolism.

### Diabetes

Diabetes mellitus, a heterogeneous disorder of glucose metabolism, has affected millions worldwide. Endothelial dysfunction is a critical and initiating factor in the genesis of diabetic vascular disease [2]. High glucose inhibits glucose phosphorylation and induces endothelial apoptosis in a mitochondria-dependent manner by suppressing HK2 expression [90, 91]. Besides high serum levels of glucose, glycated proteins and abnormal insulin release, and acid sphingomyelinase (ASM) [92] are proposed to be clinical biomarkers for diabetic vascular complication. Downregulation of ASM increases GLUT4 expression and decreases Nox2- and Nox4-dependent superoxide anion generation in cultured rat aortic endothelial cells, suggesting that high ASM levels are responsible for endothelial insulin resistance [92].

### Diabetic retinopathy

Proliferative retinopathy, presenting abnormal neovascularization with leaky, fragile, and misdirected vessels in the retina, is a characteristic feature of diabetic microvascular complications. Vitreous levels of RBP3 correlate inversely to the severity of diabetic retinopathy. An increased presence of RBP3 protein binds to GLUT1 on the endothelial cells, decreases glucose uptake, and delays the process of retinopathy [93]. Deleting GLUT1 from retinal endothelial cells reduces cell proliferation and lowers vascular outgrowth but not affecting the number of tip cells [94]. In streptozotocin-induced type 1 diabetic mice, miR-384-3p mimic reduces HK2 expression in retinal endothelial cells, resulting in decreased cell proliferation and impaired tube formation [95].

### Atherosclerosis

In apolipoprotein E^−/−^ atherosclerotic mice, enhanced glycolysis is observed in the atherosclerotic plaque-covering endothelium with impaired endothelial cell junctions. An increased amount of nanoparticles transports to the underlying extracellular matrix via the impaired endothelial barrier and later is taken up by plaque-associated macrophages [96]. Chronic inhibition of glycolysis by the small-molecule 3PO reduces plaque formation through favoring M2 macrophage phenotype but does not affect intraplaque neovascularization [97]. Taken into consideration of the protective effects of AMPK against atherosclerosis and increased glycolysis on plaque formation [see section: Shear stress and glycolysis], it suggests that AMPK activation affects other anaplerotic signalings than glycolysis.

### Cardiomyocyte dysfunction

Substantial studies have reported reduced metabolism in cardiomyocytes when subjected to ischemia/reperfusion [98–101], heat-stressed [102], or overload-induced injury [64], as well as diabetic cardiomyopathy [103]. Activation of AMPK/Akt [102, 103] or HIF-1α pathways [101], or suppression of mitochondrial fussion/mitophagy axis [99] upregulates glycolysis in endothelial cells and restores cardiac function [98, 100–102].

### Pulmonary arterial hypertension

Pulmonary arterial hypertension is characteristic of increased blood pressure in pulmonary arteries, accompanied by abnormal growth of arterioles. Increased glycolysis in endothelial cells [104, 105], underlying smooth muscle [105], and adventitial fibroblasts [106] has been observed in the hypertensive pulmonary artery. Inhibition of glycolysis by knockout PFKFB3 [105] or downregulation of PKM2 protein [104] in pulmonary endothelial cells reduces endothelial proliferation and inflammation, resulting in decreased leukocyte recruitment and smooth muscle cell proliferation. Extrinsic acidosis, but not intrinsic acidosis, inhibits glycolysis and cell migration, but improves tube formation in cultured pulmonary microvascular endothelial cells [107]. Nevertheless, in isolated pulmonary artery endothelial cells from fetal lambs with persistent pulmonary hypertension of the newborn, HIF-1α expression and its downstream glycolysis are increased, but the VEGF level and angiogenesis function are impaired. Inhibition of the HIF-1α signal downregulates the glycolytic enzymes and improves VEGF levels as well as angiogenesis [108]. It suggests that the basal and requested oxygen needed in pulmonary angiogenesis are different from adults and newborns.

### Neurological disorders

Deleting GLUT1 from postnatal brain endothelial cells reduces cell proliferation, lowers tip cell number, and blunts vascular development, whereas deleting GLUT1 in quiescent adult endothelial cells induces neuronal loss, increased inflammation, and induces severe seizures without altering BBB vascular function [94]. In a mouse Fowler syndrome model, knockout of the orphan transporter, feline leukemia virus subgroup C receptor 1, increases brain glycolysis and angiogenesis, in which vascular tips, but not CNS blood vessels, were dilated and fused [109].

### Gastroenterological disorder

In non-alcoholic fatty liver disease, liver sinusoidal endothelial cell dysfunction shows the enhanced glycolysis and increased expression of adhesion molecules, including intercellular adhesion molecule-1, platelet endothelial cell adhesion molecule-1, and E-selectin, but preserved endothelial fenestrae [110]. Knockout of prolyl hydroxylase-2, a key component in HIF signal activation, downregulates PFKFB3 expression, and accelerates hepatic steatosis in high-fat diet-fed mice [111]. Treatment with glycogen-like peptide-1 receptor agonist, exendin-4, increases endothelial glycolysis by upregulating GLUT4 expression and decelerates hepatic steatosis and fibrosis [112]. The findings indicate that increased glycolysis is a compensatory mechanism during the progression of hepatic steatosis.

Andrographolide protects gastric endothelial function by increasing PFKFB3-mediated glycolysis in an ethanol-induced gastric ulcer mouse model [113].

### Arthritis

Both psoriatic arthritis and rheumatoid arthritis are chronic inflammatory diseases characterized by swollen and stiff joints and limited joint mobility [114, 115]. Nevertheless, microvascular alterations in the joints are different. The former shows elongated and tortuous vessels with less branching, while the latter shows straight and regular branching vessels [114, 115]. In the synovium of psoriatic arthritic patients, protein expressions of glycolytic enzymes (GLUT1, PFKFB3, and PKM2) are increased in synovial vasculature, indicating a high level of glycolysis occurs [116]. HUVECs stimulated with conditioned medium of fibroblasts isolated from psoriatic arthritic patients have a higher expression of PFKFB3 and better tube formation than those stimulated with medium of fibroblasts from rheumatoid arthritic patients; the former also show a significant increase in peripheral blood mononuclear cell adhesion [116, 117].

## Therapeutic strategy

It is challenging to identify or proceed with treatments on impaired glycolysis and cellular metabolic disorders before endothelial dysfunction present or some cardiovascular symptoms develop. A healthy lifestyle is strongly recommended, including physical exercise and caloric restriction. Consistent with its protective effects in cardiovascular disease, the female hormone promotes angiogenesis. Diets rich in resveratrol content, such as peanuts, pistachios, grapes, red and white wine, blueberries, cranberries, and even cocoa and dark chocolate are suggested as well.

Physical exercise increases NO production, probably through increasing shear stress to vascular endothelial cells, which further induces mitochondrial biogenesis and glucose uptake in mouse subcutaneous adipose tissue [118]. Home-based high-intensity interval training increases muscle GLUT4 expression and improves muscle capillarization in sedentary obese adults in the United Kingdom [119].

In 24-month old rats subjected to 20% caloric restriction, endothelium-dependent relaxation is improved, and endothelium-dependent vasoconstriction is inhibited. Of importance, caloric restriction increases insulin-induced GLUT4 expression, but not basal level in the aged aorta [84]. These findings suggest that caloric restriction improves endothelial metabolism and aging-induced endothelial function.

Estradiol increases PFKFB3 expression and stimulates angiogenesis in cultured HUVEC [120, 121]; this appears to be a novel mechanism underlying estrogen protective effects on cardiovascular disease [122, 123].

Sirtuin proteins are homologs to the yeast Sir2 protein processing NAD^+^-dependent deacetylase activity. Sirtuins exert protective effects against aging, cell apoptosis, inflammatory responses, as well as energy efficiency and alertness [85, 88]. Sirtuin 3 protein is located in mitochondria and regulates metabolic switches between mitochondrial respiration and glycolysis [124, 125]. Deleting endothelial sirtuin 3 reduces GLUT1 and PFKFB3 expressions, leading to heart failure in a myocardial ischemia model and pressure overload-induced heart failure rodent models [64, 98, 125]. Activating the sirtuin 3-AMPK pathway restores pulmonary arterial pressure and vascular structure in rats with pulmonary hypertension associated with heart failure with preserved ejection fraction [126]. Polyphenolic compounds, which interact with Sirtuin molecules [127–129], reduce inflammatory responses in mice with collagen-induced arthritis through the sirtuin 6-mediated PKM2 pathway [83] and restore myocardial function through enhanced GLUT4 translocation in streptozotocin-induced type 1 diabetic rats [103]. Therefore, increased expression of sirtuin proteins, either by genetic modification or due to diet, provides beneficial effects on cell metabolism regulation. It is also reported that resveratrol restores hypoxia-induced increased expressions of GLUT1, VEGF, and leptin proteins in cultured human adipose tissue [130].

Fermented red ginseng ameliorates endothelial dysfunction, manifested with a decreased level of endothelin, reduced protein expressions of adhesion molecules, and improves glucose tolerance in muscles with increased expression of GLUT4 in rats with a high-fructose diet-induced metabolic syndrome [131].

## Conclusion

Glycolysis is the primary energy-sustaining process in endothelial cells; when the glycolytic process is compromised under pathological conditions, other metabolic processes are activated to compensate for ATP shortage, nevertheless leading to increased oxidative stress, cell dysfunction, as well as cell death. Physical exercise, caloric restriction, and diets rich in resveratrol content are recommended to boost glycolysis when cells are short of nutrients supply. However, downregulating glycolytic enzymes emerges as an important therapeutic strategy under pathological conditions since increased glycolysis and angiogenesis result in an impairment of endothelial barriers and functions. Therefore, further studies would be warranted to systemically examine the role of the different metabolic processes under different conditions, thereby providing the information on which processes/enzymes are involved in the maladaptive responses. Taken together, glycolysis and glycolytic proteins described in this review offer new insights to better characterize underlying mechanisms of endothelial dysfunction as well as cardiovascular disease and may potentially represent novel therapeutic targets to intervene in pathological angiogenesis.
